# Voxel- and tensor-based morphometry with machine learning techniques identifying characteristic brain impairment in patients with cervical spondylotic myelopathy

**DOI:** 10.3389/fneur.2024.1267349

**Published:** 2024-02-14

**Authors:** Yang Wang, Rui Zhao, Dan Zhu, Xiuwei Fu, Fengyu Sun, Yuezeng Cai, Juanwei Ma, Xing Guo, Jing Zhang, Yuan Xue

**Affiliations:** ^1^Department of Radiology, Tianjin Medical University General Hospital, Tianjin, China; ^2^Tianjin Key Laboratory of Functional Imaging, Tianjin Medical University General Hospital, Tianjin, China; ^3^Department of Orthopedics Surgery, Tianjin Medical University General Hospital, Tianjin, China; ^4^Department of Radiology, Tianjin Medical University General Hospital Airport Hospital, Tianjin, China; ^5^Tianjin Key Laboratory of Spine and Spinal Cord, Tianjin Medical University General Hospital, Tianjin, China

**Keywords:** cervical spondylotic myelopathy, structural MRI, tensor-based morphometry, voxel-based morphometry, multivariate pattern analysis

## Abstract

**Aim:**

The diagnosis of cervical spondylotic myelopathy (CSM) relies on several methods, including x-rays, computed tomography, and magnetic resonance imaging (MRI). Although MRI is the most useful diagnostic tool, strategies to improve the precise and independent diagnosis of CSM using novel MRI imaging techniques are urgently needed. This study aimed to explore potential brain biomarkers to improve the precise diagnosis of CSM through the combination of voxel-based morphometry (VBM) and tensor-based morphometry (TBM) with machine learning techniques.

**Methods:**

In this retrospective study, 57 patients with CSM and 57 healthy controls (HCs) were enrolled. The structural changes in the gray matter volume and white matter volume were determined by VBM. Gray and white matter deformations were measured by TBM. The support vector machine (SVM) was used for the classification of CSM patients from HCs based on the structural features of VBM and TBM.

**Results:**

CSM patients exhibited characteristic structural abnormalities in the sensorimotor, visual, cognitive, and subcortical regions, as well as in the anterior corona radiata and the corpus callosum [*P* < 0.05, false discovery rate (FDR) corrected]. A multivariate pattern classification analysis revealed that VBM and TBM could successfully identify CSM patients and HCs [classification accuracy: 81.58%, area under the curve (AUC): 0.85; *P* < 0.005, Bonferroni corrected] through characteristic gray matter and white matter impairments.

**Conclusion:**

CSM may cause widespread and remote impairments in brain structures. This study provided a valuable reference for developing novel diagnostic strategies to identify CSM.

## Introduction

Cervical spondylotic myelopathy (CSM) refers to a neurological disorder characterized by spinal cord compression caused by degenerative changes in the cervical spine. An epidemiological survey reported that the global incidence of CSM has been increasing annually as a result of population aging (1), making it the most common cause of spinal cord injury (SCI) worldwide (accounting for 54% of non-traumatic spinal cord injuries) (2). Therefore, it is necessary to conduct physical examinations for patients with suspected CSM. However, the symptoms of CSM are almost always insidious (3), which makes the diagnosis of CSM very challenging (4). Currently, imaging is still an important approach for evaluating suspected CSM.

Magnetic resonance imaging (MRI) has been considered the preferred imaging technique for diagnosing CSM, which allows for a specific assessment of the compression severity of the spinal cord (2, 5). The presence of T2 hyperintensity and/or T1 hypointensity in the spinal cord can serve as diagnostic indicators of CSM. Nevertheless, these features have an unsatisfactory sensitivity of 15%−65% and are not present in all patients with clinical signs (6, 7). Functional MRI and diffusion tensor imaging (DTI) studies have focused on the impacts of CSM on the brain (8–13). However, these studies were mostly based on univariate analyses, and their diagnostic value for CSM has not been further confirmed. Currently, morphometric studies based on MRI, including voxel-based morphometry (VBM) and tensor-based morphometry (TBM), are recognized as highly effective methods for revealing the neurological and psychological substrates of a disease (14–16). Furthermore, a multivariate pattern analysis (MVPA) of MRI data provides an unprecedented possibility to detect subtle differences in the spatial patterns of structural brain changes and reorganization in the brain between sick and healthy individuals (16, 17). The support vector machine (SVM) is a type of MVPA technique with high accuracy in the discrimination of binary classification (18). Fernandez Rojas et al. (18) used the SVM algorithm with 25 features to identify the four types of pain with a 94.17% accuracy. Previous studies reported that the SVM technique has much potential to identify features from different brain parts that can be used to classify healthy controls (HCs) and CSM patients (16, 19).

In this study, to develop a model with clinically potential diagnostic properties for CSM patients, we tested the utility of structural MRI as a potential biomarker for CSM patients using the SVM. Then, the diagnostic performance of SVM, extreme gradient boosting (XGBoost), and light gradient boosting machines (LightGBM) was further explored. Structural changes in CSM patients were first estimated by a univariate analysis of VBM and TBM at the whole-brain level. Then, MVPA was adopted to classify CSM patients and HCs based on the morphological information extracted by VBM and TBM via SVM. Finally, separate MVPAs were performed for each brain region of the whole brain based on the template to validate the potential biomarkers for the diagnosis of CSM.

## Methods

### Participants

In this retrospective study, 60 right-handed CSM patients were continuously recruited at Tianjin Medical University General Hospital, Tianjin, China from 2015 to 2021. The inclusion criteria were as follows: (a) CSM patients who had spinal cord compression identified by cervical spine MRI; (b) patients with hyperreflexia, extremity spasticity, intrinsic muscle atrophy, Hoffmann reflexes, hand stiffness, or gait dysfunction; (c) patients with clinical manifestations of extremity sensorimotor deficits or bladder and bowel dysfunction; and (d) patients with complete MRI examinations. The exclusion criteria were as follows: (a) patients with a history of cervical spinal surgery; (b) patients with stenosis of the extracranial portion of the vertebral artery or in the carotid artery after Doppler ultrasound examination; (c) patients with clinical evidence or a history of other neurological, psychiatric, or systemic diseases, including hypertension, diabetes, active infection, neoplastic disease, rheumatoid arthritis, and ankylosing spondylitis after consultation with specialists; (d) patients with white matter hyperintensity lesions and lacunar infarction in MRI images; (e) patients with high-energy trauma fractures; (f) patients with metastatic fractures; (g) patients with concomitant lumbar stenosis; and (h) patients with alcohol or substance abuse. All eligible CSM patients underwent clinical examination and evaluation using the Japanese Orthopedic Association (JOA) Scoring System scale before MRI to assess neurological dysfunction due to spinal cord compression.

A total of 60 right-handed HCs with matching age, sex, and education were actively recruited. The inclusion criteria were as follows: (a) participants without spinal compression identified by cervical spine MRI; (b) participants without other spinal or brain neurological disorders or systemic disease; and (c) participants with complete MRI examinations. This study was approved by the institutional review board of Tianjin Medical University General Hospital, Tianjin, China (No. IRB2023-WZ-065). Written informed consent was provided by all participants.

### Clinical examination

All CSM patients underwent clinical examination and evaluation with the JOA Scoring System scale before MRI to assess neurological dysfunction due to spinal cord compression.

### MRI data acquisition and image preprocessing

**Figure 1:**
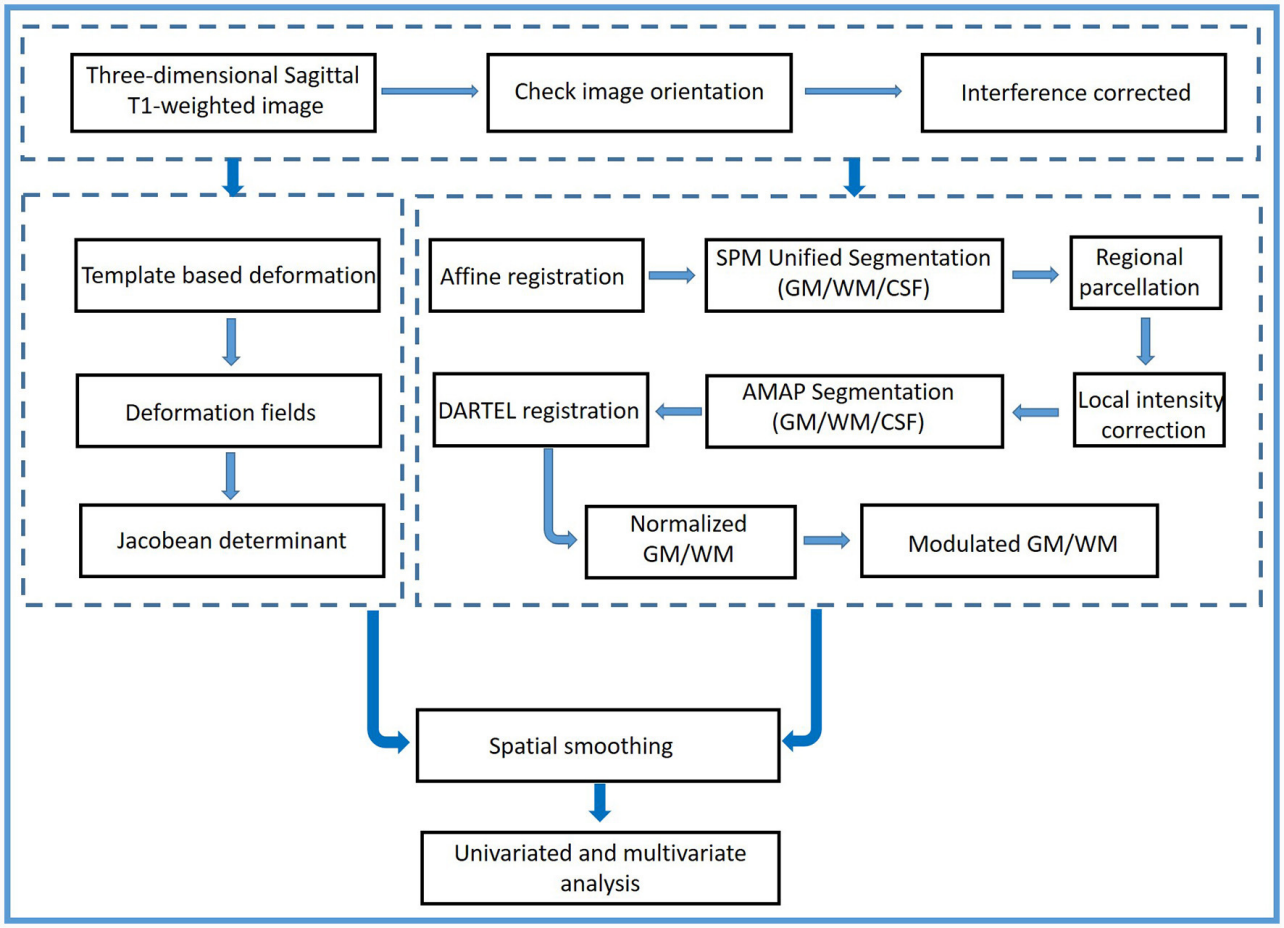
The flowchart of the methodology.

The flowchart of the methodology is shown in Figure 1. Using two 3.0-Tesla MRI scanners [the Discovery MR750 scanner (GE Healthcare, VE 11C, Milwaukee, WI, United States) and the Prisma scanner (Siemens Healthineers, DV24.0_R01_1344.a, Erlangen, Germany)] from Tianjin Medical University General Hospital, three-dimensional sagittal T1-weighted images were obtained. The imaging parameters of the MR750 scanner were as follows: repetition time/echo time = 7.8/3.0; inversion time = 450 ms; flip angle = 13°; thickness = 1.0 mm; field of view = 256 × 256; matrix = 256 × 256; no gap; and 180 sections. The imaging parameters of the Prisma MR scanner were as follows: repetition time/echo time = 4,000/2.32; flip angle (FA1/FA2) = 4°/5°; TI1/TI2 = 700/2,110; thickness = 1.0 mm; field of view = 256 × 240; matrix = 256 × 240; no gap; and 208 sections. All images had the same spatial resolution (1 × 1 × 1 mm^3^).

First, all images were double-checked by an experienced radiologist (Y Wang) to ensure that they had the same orientation parallel to the anterior–posterior commissure line. Then, a two-step procedure before preprocessing was performed. The bias field was corrected for all images. The local and global intensity were normalized, while image inhomogeneities and noise were removed using a spatial adaptive non-local means filter. The strength of graph-cut-based skull stripping was 0.5. The processed structural images were preprocessed using the CAT12 toolbox (Computational Anatomy Toolbox; http://www.neuro.uni-jena.de, Jena, Germany) and Statistical Parametric Mapping software (SPM12, https://www.fil.ion.ucl.ac.uk/spm/software/spm12, Functional Imaging Laboratory, UCL Queen Square Institute of Neurology, London, UK).

### VBM and TBM assessment

The processed images were segmented into the gray matter (GM), the white matter (WM), and the cerebrospinal fluid (CSF) by integrating the Markov random field and adaptive maximum (a posterior segmentation technique) (20). Then, the affine and non-linear co-registration of GM and WM images to the normalized Montreal Neurological Institute (MNI) space was performed using diffeomorphic anatomic registration through the exponentiated Lie algebra technique (DARTEL) and the geodesic shooting normalization algorithm (21, 22). The structural images were registered at 1.5 mm^3^ resolution. Subsequently, a local volume assessment was performed to identify the mixed tissue types, including GM-WM and GM-CSF combinations (23). To check the quality of segmented images, the single slices were visualized, and the GM and WM modifications were proportionally scaled. Then, modulated normalization GM volume images (GM-VBM) and WM volume images (WM-VBM) were generated. Sample homogeneity was checked utilizing these modulated normalized segments via boxplots and correlation matrices, and the correlation coefficients < 0.83 were removed. Finally, the GM-VBM and WM-VBM images were smoothed with a full width at a half-maximum (FWHM) kernel of 6 mm. The smoothed GM-VBM and WM-VBM images were used for a statistical group analysis. Moreover, the TIV for each subject was calculated as the equivalence to an estimation of the sum of GM, WM, and CSF values by CAT12.

The deformation fields were obtained using a three-dimensional non-linear registration approach when the template was applied to each processed individual structural image. The deformation information was coded in deformation fields. The resultant voxel-wise deformation fields represented the three-dimensional transformations resulting from the local deformation of each brain structure to match the template. The deformation information in the deformation fields included differences in positional displacement and local brain size. Consequently, deformed maps of local voxel-wise changes were quantified by the Jacobian determinant. The deformed maps were used as a measure of tissue shrinkage or expansion (24). Subsequently, the deformed map of each brain was spatially smoothed by a 6-mm FWHM. The smoothed TBM was used for group statistical analysis. TBM Jacobian determinant maps were divided into GM-TBM images and WM-TBM images by using the GM and WM masks, respectively.

### Univariate statistical analysis

The intergroup differences in GM/WM-VBM and GM/WM-TBM between the CSM and HC groups were evaluated using two-sample *t*-tests controlling for age, sex, education level, and scanners. The TIV of each subject was regarded as a covariate in the GM/WM-VBM statistical analysis (25). All results were corrected for multiple comparisons using the false discovery rate (FDR) at the cluster level (the initial height threshold: uncorrected *P* < 0.001; topological FDR: *P* < 0.05) (26). When comparing VBM with TBM, the overlapping results between the corrected GM/WM-VBM and GM/WM-TBM maps from the GM/WM results at the volumetric level were analyzed.

### Correlation analysis

The clusters with significant differences in VBM and TBM results were identified. Statistically significant cluster peak coordination as the center and 3 mm as the radius were used to generate spheres. The average variability in the sphere was extracted. Spearman's correlation analyses were conducted between significant clusters and preoperative JOA scores with age, sex, education level, and scanners as covariates. A *P-*value of < 0.05 indicated a significance threshold.

### Multivariate pattern classification analysis

MVPA was performed on each measurement method (GM-VBM, WM-VBM, GM-TBM, and WM-TBM) to distinguish CSM patients from HCs. The analysis was performed via the MVPANI toolbox (https://github.com/pymnn/MVPANI) (27), which was combined with LibSVM to implement a linear SVM (28). Using leave-one-fold-out cross-validation, MVPA was performed. The MVPA processing procedures were consistent for each measurement method and are described in the Supplementary material. A permutation test was used to test the statistical significance of the cross-validation. The *P*-value with statistical significance was calculated based on the null distribution obtained from permutation tests (1,000 random times) and was corrected for multiple comparisons. If none of the 1,000 permutations reached the actual accuracy value, the *p*-value was reported as being < 0.001. In this procedure, 10 independent MVPAs were performed. Thus, the *p*-values were further corrected for multiple comparisons by the Bonferroni correction (*P* < 0.05/10 = 0.005).

To identify whether different structural measurement methods provided complementary information and improved the classification accuracy, a procedure for data fusion was performed using a weighted voting strategy based on the classification results from all measurement methods (GM-VBM, WM-VBM, GM-TBM, and WM-TBM). The MVPA process for data fusion is described in the Supplementary material. According to the results of the chance-level classification, the null distribution after fusion was calculated during the permutation tests, and the *p*-value was obtained. This fusion procedure was performed for each feature selection percentage from permutations (1,000 random times) and was corrected for multiple comparisons by the Bonferroni correction (*P* < 0.005).

### Validation analysis

MVPA based on various brain regions at the whole brain level was performed to confirm that the characteristic brain areas were statistically significant in the accuracy of categorization of CSM patients and HCs. Different brain regions were defined as the regions of interest (ROIs). Human brain atlases and WM atlases were used as the mask in GM and WM ROI-based MVPA (29, 30). The cross-validation technique was implemented under the same conditions as for MVPA using a single structural measurement method. The *p*-value was calculated based on the null distribution obtained from permutation tests (1,000 random times). The *p*-value from the permutation tests was corrected for multiple comparisons using the FDR correction.

## Results

### Demographic data and clinical evaluation

A total of 60 CSM patients and 60 HCs participated in this study. Furthermore, six subjects were excluded due to incomplete coverage of the whole brain during imaging (1 out of 6, 17%), motion artifacts in structural images (3 out of 6, 50%), and post-processing findings that conflicted with the requirement (within two standard deviations) of VBM for variance inhomogeneity (2 out of 6, 33%). A total of 57 CSM patients (23 women, 34 men, 52.7 ± 12.4 years) and 57 HCs (26 women, 31 men, 51.0 ± 13.4 years) were finally included in the analysis. Data acquired using the MR750 scanner from 27 CSM patients and 11 HCs as well as the data from the Prisma MR scanner from 30 CSM patients and 46 HCs were included.

The demographic data of the CSM and HC groups are shown in Supplementary Table S1. No significant differences in age, sex, or education level were observed between the CSM and HC groups (all *P* > 0.05). The preoperative JOA scores of the CSM groups are also shown in Supplementary Table S1. There were no significant differences in JOA scores between the data of CSM patients obtained from the MR750 and Prisma scanners (*P* = 0.310).

### Univariate pattern analysis

#### Reduced GM and WM volume in CSM patients based on VBM analysis

**Figure 2:**
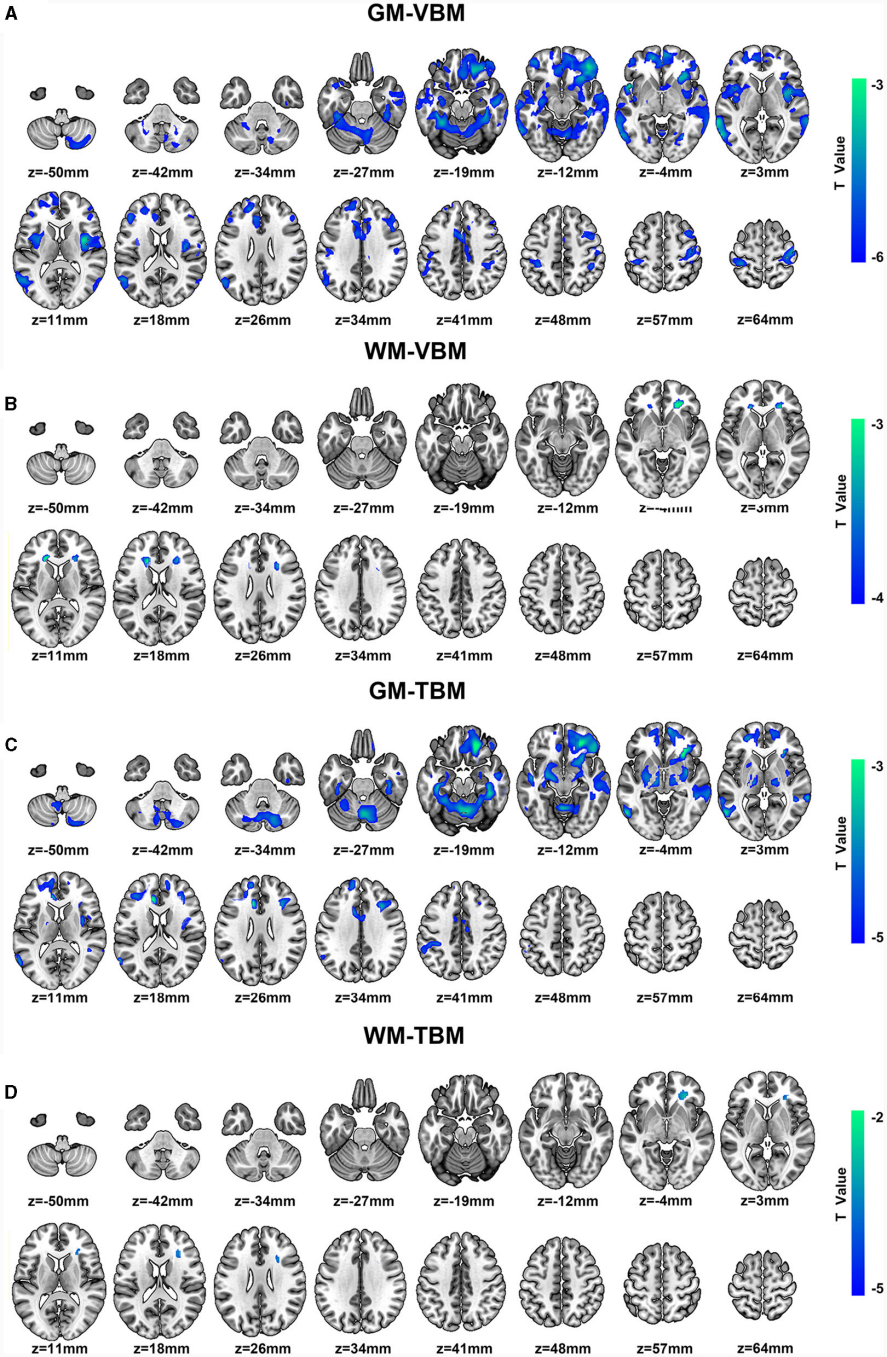
**(A, B)** Regional GM volume and WM volume changes detected by VBM and color bar for clusters (*P* < 0.05, FDR corrected). **(C, D)** Regional deformation of GM and WM detected by TBM and color bar for clusters (*P* < 0.05, FDR corrected). All regions indicated in colors ranging from blue to green (assigned according to *t* values from two-sample *t*-tests) have reduced volumes in CSM patients compared with those in HCs.

Significant GM volume loss was observed in the bilateral primary sensorimotor cortex (SM1), basal ganglia (BG) (pars putamen, pars right caudate, and pars right amygdala), right premotor cortex, right parietal operculum (OP), and cerebellum of CSM patients (Figure 2A, Supplementary Table S2). In addition, reduced GM volume was also observed in the bilateral superior frontal gyrus (SFG) (pars medial and orbital), middle frontal gyrus (MFG), inferior frontal gyrus (IFG) (pars orbital, opercular, and triangular), fusiform gyrus, middle cingulate cortex (MCC), insula, rolandic operculum (ROL), lingual gyrus and hippocampus, unilateral middle occipital gyrus (MOG), anterior cingulate cortex (ACC) on the left hemisphere and unilateral middle orbitofrontal cortex, and gyrus rectus (REC) on the right hemisphere in CSM patients (Figure 2A, Supplementary Table S2).

Moreover, significantly reduced WM volume was located on the bilateral anterior corona radiata (ACR) and the right side of the corpus callosum (CC) (pars genu and body) (Figure 2B, Supplementary Table S3). In addition, no significant increases in GM or WM volumes were noted in the whole brain.

#### Morphological deformation of GM and WM in CSM patients based on TBM analysis

Significant deformation of GM-TBM was obvious in the bilateral SFG (pars medial and orbital), MFG, IFG (pars triangular), REC, fusiform gyrus, MCC, hippocampus, thalamus, BG (pars amygdala, pars putamen, pars left pallidum, and pars right caudate), cerebellum, unilateral primary somatosensory cortex (S1), and ACC on the left hemisphere of CSM patients as well as in the unilateral middle orbitofrontal cortex, inferior orbitofrontal cortex, ROL, insula, and para-hippocampus on the right hemisphere of these patients (Figure 2C, Supplementary Table S4). Significant structural deformation based on WM-TBM was located on the right ACR (Figure 2D, Supplementary Table S5). No significant expansion of GM-TBM or WM-TBM was noted in the whole brain.

#### GM changes detected by VBM and TBM

**Figure 3:**
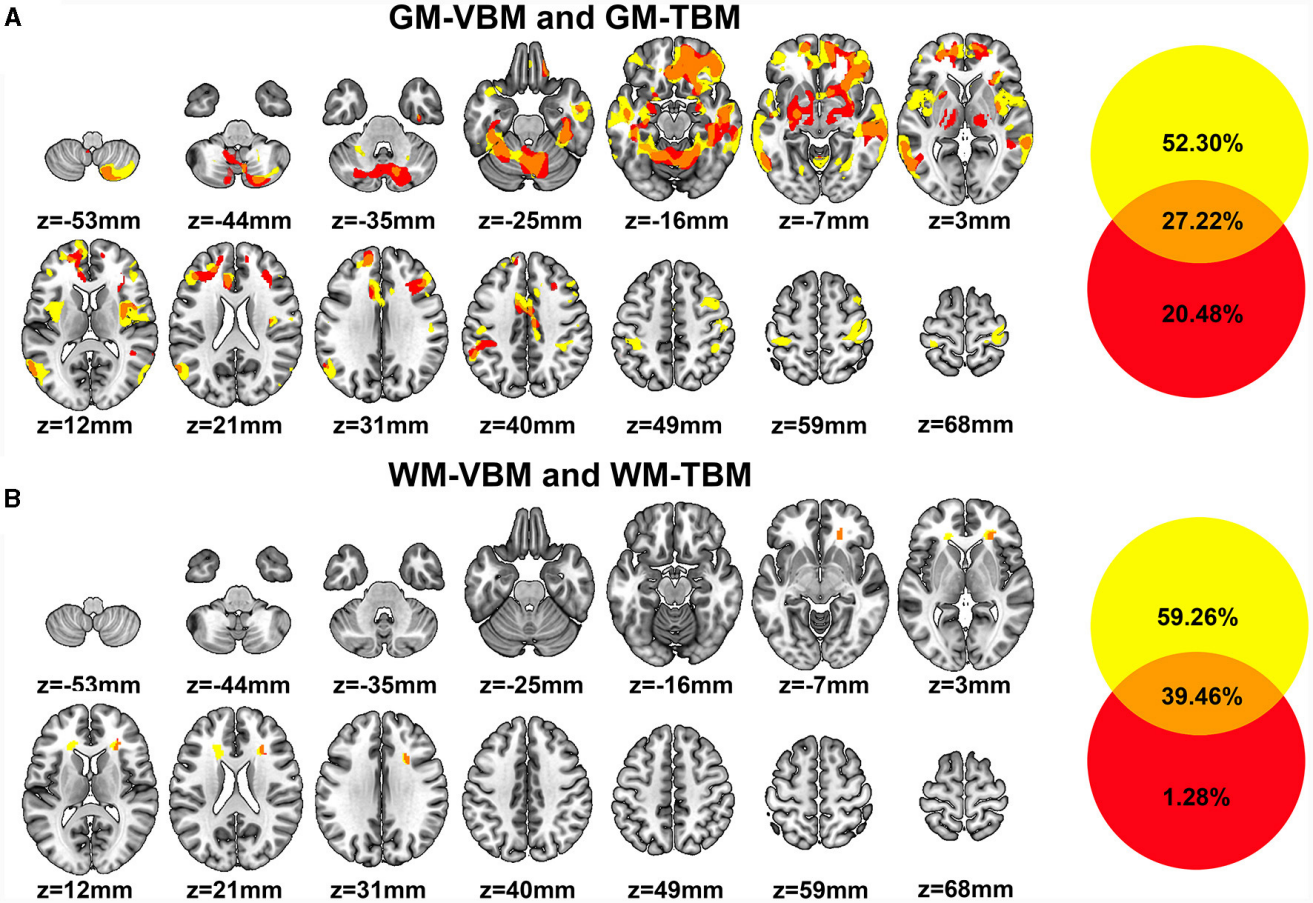
**(A)** Regions of GM structural differences in CSM patients compared to those in HCs detected by VBM (GM-VBM, in yellow) and TBM (GM-TBM, in red) are shown in volumetric space. The overlapping regions of GM-VBM and GM-TBM are shown in orange. **(B)** Regions of WM structural differences in CSM patients compared to those in HCs detected by VBM (WM-VBM, in yellow) and TBM (WM-TBM, in red) are shown in volumetric space. The overlapping regions of WM-VBM and WM-TBM are shown in orange.

There were extensive overlapping regions of GM structural alterations across VBM and TBM identified in CSM patients. Of the GM structural alterations identified by at least one of the two measurement methods, 27.22% of GM structural alterations were detected by both VBM and TBM. Overlapping GM accounted for 34.23% of the total GM-VBM and 57.07% of the total GM-TBM. These overlapping regions included the bilateral SFG (pars medial and orbital), MFG, IFG triangular, fusiform gyrus, MCC, hippocampus, BG (bilateral putamen, right caudate, right amygdala) cerebellum, unilateral S1, and ACC on the left hemisphere, as well as in the middle orbitofrontal cortex, inferior orbitofrontal cortex, ROL, insula, and REC on the right hemisphere (Figure 3A).

In addition, several regions of GM structural alterations were detected by either VBM or TBM. A total of 52.30% of GM structural alterations were detected by VBM alone (Figure 3A), including the bilateral primary motor cortex (M1), inferior frontal opercular and lingual gyrus, and unilateral MOG in the left hemisphere. Compared with VBM, 20.48% of the total GM structural alterations were detected by TBM alone (Figure 3A). Deformation in some subcortical regions, including the bilateral thalamus, BG, and right para-hippocampus, was detected by TBM alone (Figure 3A).

#### WM changes detected by VBM and TBM

We further investigated the overlapping regions of the WM structural alterations detected by VBM and TBM. The overlap rate of WM structural changes accounted for 39.46% of the total volume alterations detected by WM-VBM and WM-TBM. The overlapping regions of WM alterations accounted for 39.97% of the total WM-VBM and 96.85% of the total WM-TBM. The WM volume reductions determined by VBM were confined to the bilateral ACR and the right side of the CC (Figure 3B). TBM only detected WM deformation in the right ACR (Figure 3B). The structural alterations of WM in the right ACR widely overlapped with the changed WM regions detected by both VBM and TBM (Figure 3B). The non-overlapping ratio was 59.26% for WM-VBM and 1.28% for WM-TBM.

### Correlation analysis

**Figure 4:**
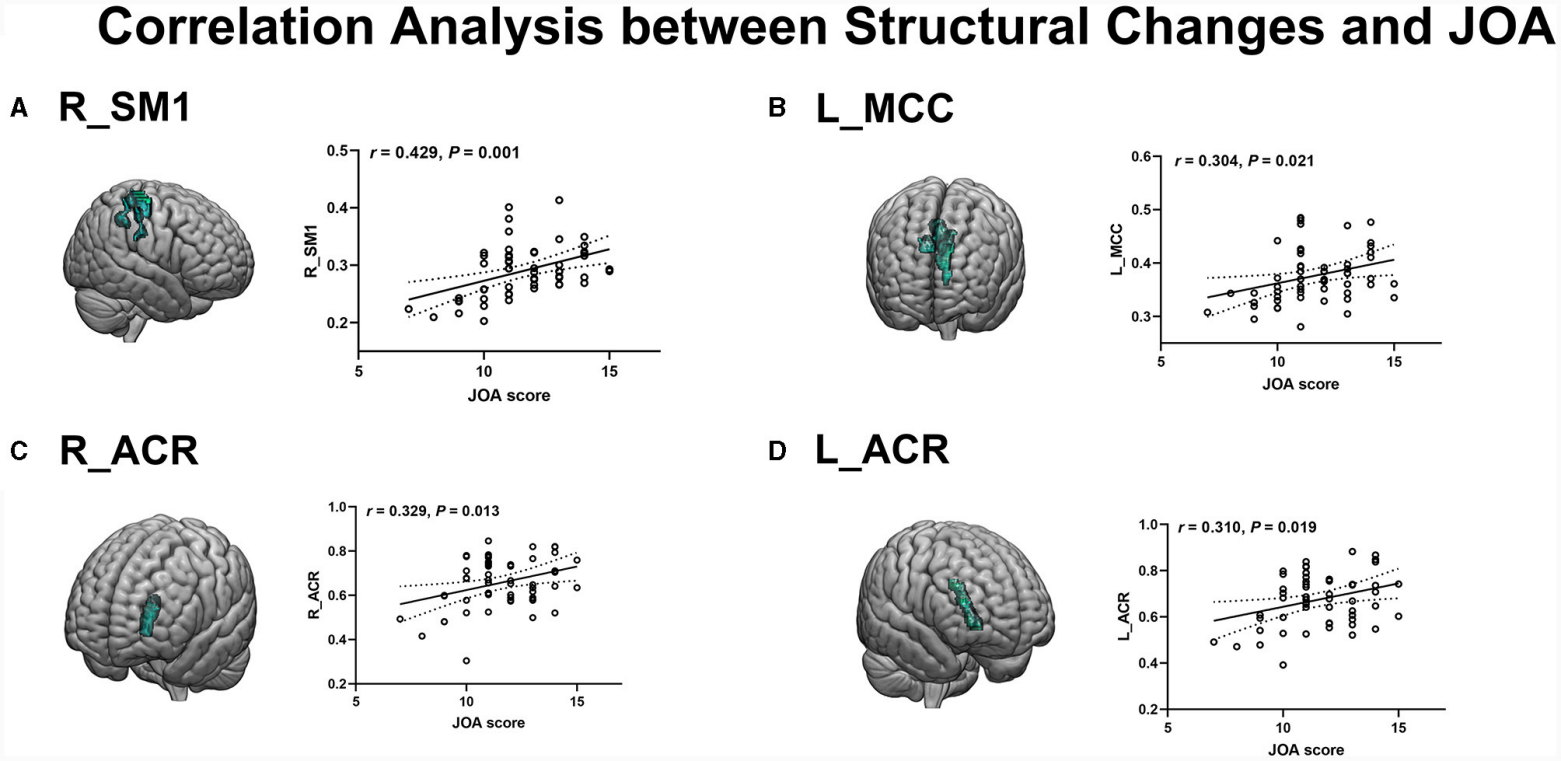
**(A, B)** Positive correlation of reduced GM in the R_SM1 and L_MCC with JOA scores. **(C, D)** Positive correlation of reduced WM in R_ACR and L_ACR with JOA scores.

The JOA score of CSM patients was positively correlated with the reduced GM volume (measured by GM-VBM) in the right SM1 (*r* = 0.429, *P* = 0.001) and the left MCC (*r* = 0.304, *P* = 0.021) (Figures 4A, B). In addition, a positive correlation was also observed between the JOA score and the reduced WM volume (measured by WM-VBM) in the bilateral ACR (right: *r* = 0.329, *P* = 0.013, left: *r* = 0.310, *P* = 0.019) (Figures 4C, D).

### Multivariate pattern classification analysis

#### Classification analysis at the whole-brain level

**Figure 5:**
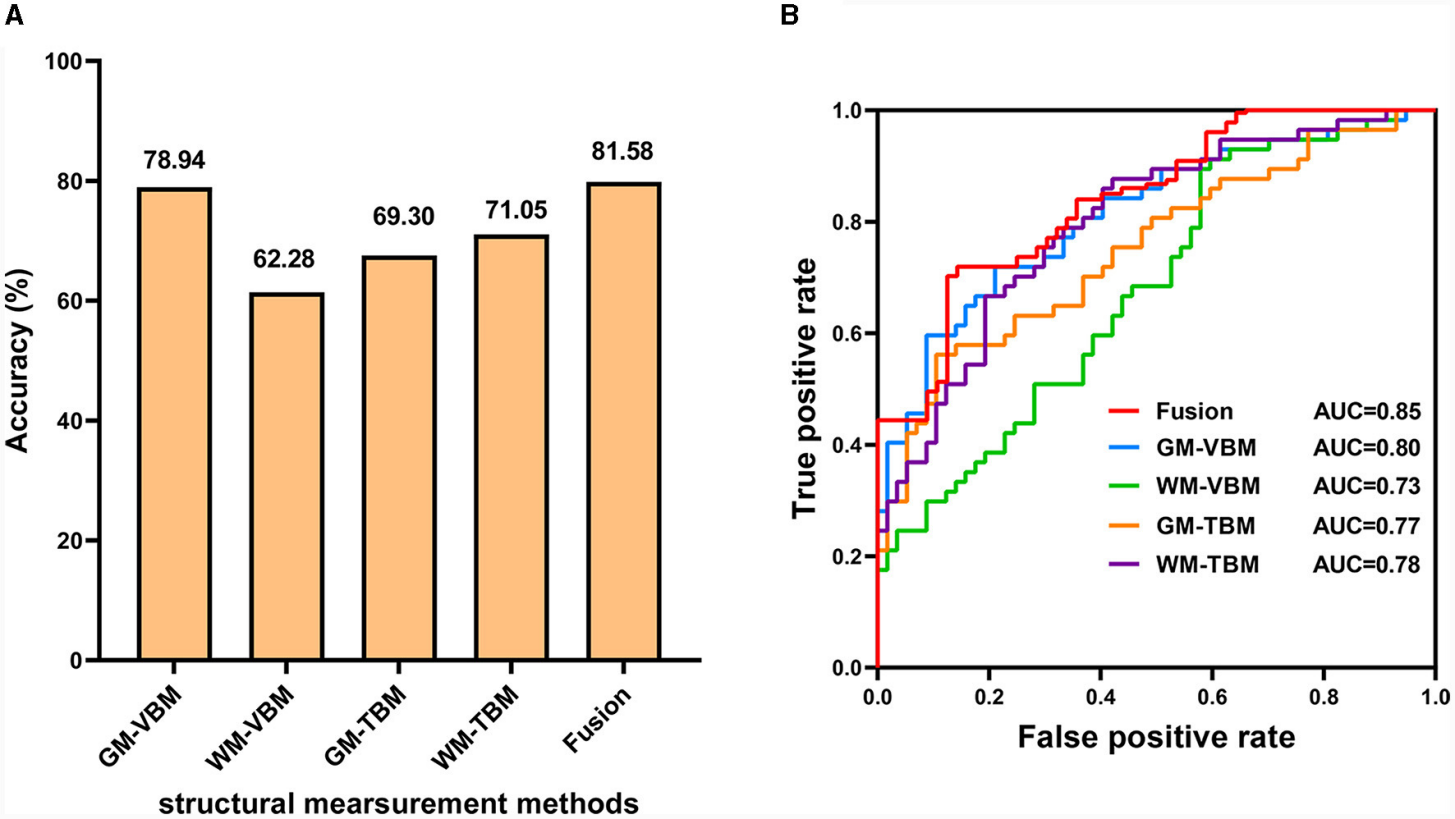
The diagnostic performance of the SVM model. **(A)** Classification accuracy for each structural measurement method and combined measurement method (fusion). **(B)** The ROC curves for each structural measurement method and the combined measurement method (fusion).

The number of feature selections corresponding to the highest classification accuracy for VBM and TBM was 10% for GM-VBM, 10% for WM-VBM, 20% for GM-TBM, and 30% for WM-TBM. The highest average classification accuracy across samples of each measurement method was 78.94% for GM-VBM, 62.28% for WM-VBM, 69.30% for GM-TBM, 71.05% for WM-TBM, and 81.58% for the combined four measurement methods (fusion) (Figure 5A). The combined four measurement methods acquired higher classification accuracies compared to the single measurement method. The weight map for the feature selection corresponding to the highest classification accuracy is shown in Supplementary Figure S1. As a result of the permutation tests, the classification accuracy of the four measurement methods and the fusion method was significantly better than chance (*P* < 0.005; Supplementary Figure S2). The area under the curve (AUC) values of the receiver operating characteristic (ROC) curves for VBM and TBM were 0.80 for GM-VBM, 0.73 for WM-VBM, 0.77 for GM-TBM, 0.78 for WM-TBM, and 0.85 for the combined features (Figure 5B). MVPA results without scanner differences as covariates are shown in the Supplementary Figures S3–S5.

**Figure 6:**
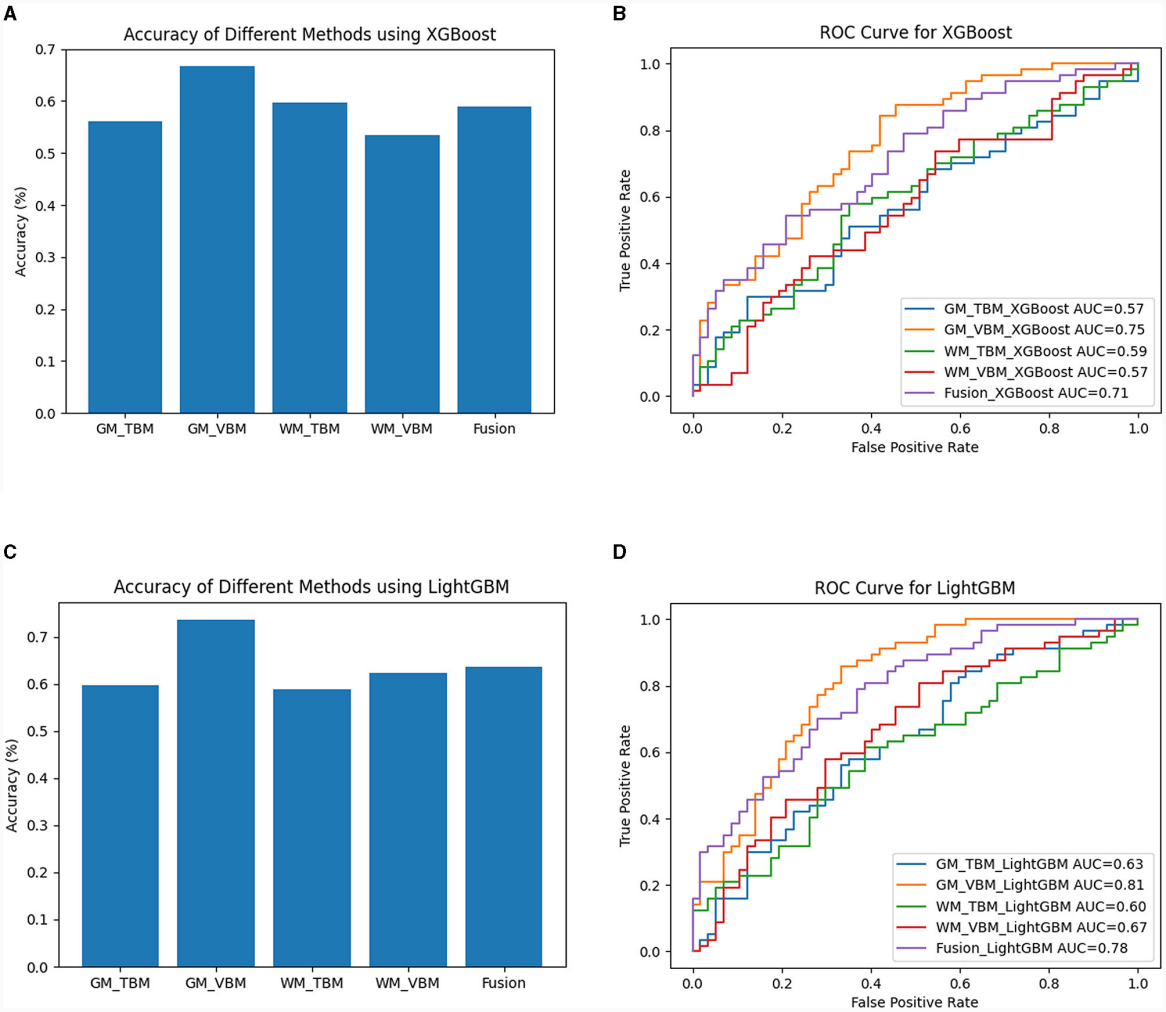
The diagnostic performance of the XGBoost and LightGBM models. **(A)** Classification accuracy for each measurement method and the combined measurement method (fusion) by XGBoost. **(B)** The ROC curves for each structural measurement method and the combined measurement method (fusion) by XGBoost. **(C)** Classification accuracy for each measurement method and the combined measurement method (fusion) by LightGBM. **(D)** The ROC curves for each structural measurement method and the combined measurement method (fusion) by LightGBM.

For XGBoost analysis, the highest average classification accuracy across samples of each measurement method was 56.14% for GM-TBM, 66.67% for GM-VBM, 59.65% for WM-TBM, 53.51% for WM-VBM, and 58.99% for the combined four measurement methods (fusion) (Figure 6A). The AUC values of VBM and TBM were 0.75 for GM-VBM, 0.57 for WM-VBM, 0.57 for GM-TBM, 0.59 for WM-TBM, and 0.78 for the combined features (Figure 6B). For LightGBM analysis, the highest average classification accuracy across samples of each measurement method was 59.65% for GM-TBM, 73.69% for GM-VBM, 58.77% for WM-TBM, 62.28% for WM-VBM, and 63.60% for the combined four measurement methods (fusion) (Figure 6C). The AUC values of the ROC curves for VBM and TBM were 0.81 for GM-VBM, 0.67 for WM-VBM, 0.63 for GM-TBM, 0.60 for WM-TBM, and 0.78 for the combined features (Figure 6D). Overall, the accuracy of XGBoost and LightGBM was not superior to that of SVM. The AUC values of the GM-VBM calculated by LightGBM were only slightly higher than the results obtained by SVM.

### Validation analysis

**Figure 7:**
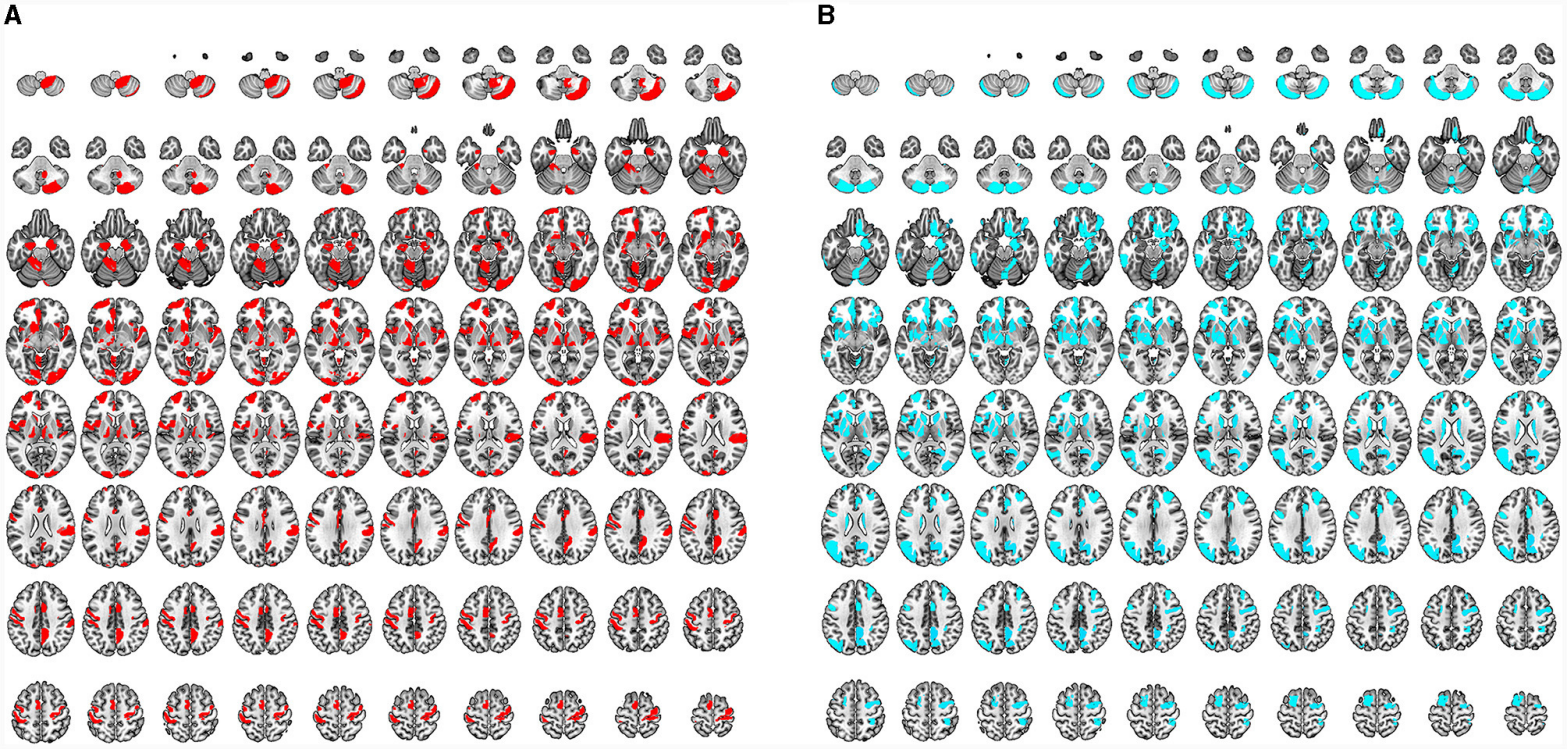
**(A)** Characteristic regions for distinguishing CSM patients from HCs as detected by VBM. All regions indicated in red are the characteristic regions for the diagnosis of CSM (corresponding to significant accuracy of classification analysis; permutation test, *P* < 0.05, FDR corrected). **(B)** Characteristic regions for distinguishing CSM patients from HCs as detected by TMB. All regions indicated in blue are the characteristic regions for the diagnosis of CSM (corresponding to significant accuracy of classification analysis; permutation test, *P* < 0.05, FDR corrected).

**Figure 8:**
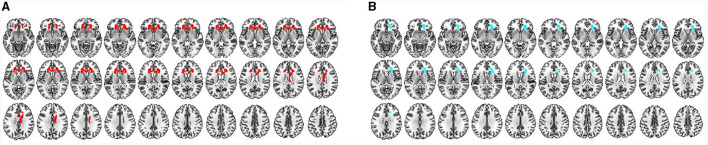
**(A)** Characteristic WM regions for distinguishing CSM patients from HCs as detected by VBM. All regions indicated in red are the characteristic regions for the diagnosis of CSM (corresponding to significant accuracies from classification analysis; permutation test, *P* < 0.05, FDR corrected). **(B)** Characteristic WM regions for distinguishing CSM patients from HCs as detected by TMB. All blue regions are the characteristic regions for the diagnosis of CSM (corresponding to significant accuracies from classification analysis; permutation test, *P* < 0.05, FDR corrected).

Based on the brain regions in MVPA, some shared brain regions that distinguished CSM patients from HCs were detected in VBM and TBM, including the premotor area, SM1, prefrontal cortex (PFC), insula, precuneus, hippocampus, BG (medial amygdala and ventral caudate), thalamus, cerebellum (crus II, V), and right ACR (Figures 7, 8).

Differential brain regions were also discovered by either VBM or TBM. In GM ROI-based MVPA of VBM, the main differential regions included the ACC, occipital cortex [occipital pole, inferior occipital gyrus (IOG), and medioventral occipital cortex], BG (nucleus accumbens), and cerebellum (I–IV, VIIIb, and IX). In ROI-based MVPA of TBM, the main differential regions included the MOG, BG (globus pallidus, dorsolateral putamen, and dorsal caudate), and cerebellum (Vermis VI) (Figure 7). WM ROI-based MVPA of VBM detected more characteristic regions than TBM, including the left ACR, bilateral genu of the CC, and right body of the CC (Figure 8). The detailed results of MVPA based on different brain regions are displayed in the Supplementary Table S6.

## Discussion

In the present study, we investigated the characteristic brain structural alterations in CSM patients with VBM and TBM. Furthermore, we evaluated the discriminative capability of brain structural alterations to diagnose CSM and validated the characteristic brain regions associated with CSM by MVPA. The main results were as follows. (1) The characteristic structural changes in CSM involved the brain regions responsible for multiple functions, including not just the sensorimotor areas but also the visual and cognitive areas. These regions were also associated with CSM classification accuracy. (2) In GM, VBM was more sensitive to the detection of structural alterations in CSM patients. Structural alterations detected by VBM and TBM were more similar in WM than in GM. (3) The alterations of the brain structure in CSM patients were specific to both VBM and TBM. The CSM diagnostic accuracy was improved using VBM, TBM, and a combination of both measurement methods. These findings provide novel neuroimaging biomarkers for the identification of CSM and offer greater possibilities for the accurate diagnosis and treatment of CSM. In addition, our findings may be alternative targets for improving brain plasticity in CSM, which may provide valuable information for developing novel diagnosis and rehabilitation therapies after postoperative decompression of CSM.

### Characteristic GM changes in the CSM group correlated with the JOA score

The present study found extensive GM volume reduction and deformation in the brain regions associated with sensorimotor, visual, cognitive, and pain modulation functions. Furthermore, the reduced GM volume in the SM1 and MCC was positively correlated with the neurological dysfunction measured by the JOA score. In addition, the regions associated with sensorimotor, visual, and cognitive functions were confirmed to distinguish CSM patients from HCs in MVPA.

In our study, the GM volume reduction and deformation of sensorimotor regions were located in the SM1 region, premotor cortex, OP, BG, thalamus, and cerebellum (31), which was consistent with previous structural and fMRI studies (31–33). However, the mechanisms of GM volume reduction and deformation in sensorimotor-associated regions remained unclear. Studies on SCI recognized that the focal lesions of the spinal cord caused a reorganization of the sensorimotor cortex, thalamus, BG, and cerebellum (34–38). CSM and SCI may have similar characteristics in terms of structural changes in cortical areas associated with the sensorimotor function. More importantly, the characteristic sensorimotor regions, including the hubs of the sensorimotor network (SMN), were useful for the classification of CSM patients and HCs. Our ROI-based MVPA results suggested that the changes in the hubs of the SMN network were the characteristic regions of CSM diagnosis.

The present study observed that the visual correlation regions with the GM volume reduction and deformation included the lingual gyrus, fusiform gyrus, and MOG in CSM. An fMRI study reported decreased neural activities in the visual cortex in CSM patients (39). Our structural study supplemented the discoveries of the functional studies of the visual cortex. The reduced GM volume may relate to decreased local neural activities in the occipital lobe. Our ROI-based MVPA results also involved the visual network (VN), indicating that VN variations could be used as distinct regions to improve the diagnostic accuracy of CSM.

Compared with HCs, the GM volume reduction and deformation located in the PFC were found in the CSM group, including the SFG, MFG, and orbitofrontal cortex (OFC). The PFC was associated with working memory, rule learning, planning, attention, motivation, and execution of the function (40–43). Several studies have also revealed that connectivity exists between the PFC and the primary sensory cortices as well as the occipital lobes (9, 44–48). Moreover, a neurological study showed that axons from the orbitofrontal and medial prefrontal cortices had neurons projected to the spinal cord via the hypothalamus (49). Therefore, according to the results of our study, the atrophic PFC in CSM may be associated with the disintegration of axons and a reduction in the dendritic density of the motor cortex, visual cortex, thalamus, cerebellum, and spinal cord. In addition, our ROI-based MVPA results demonstrated that cognitive networks, including the central executive network (CEN) (PFC, ACC, and parietal cortex), salience network (SN) (insula), and default-mode network (DMN) (PFC, ACC, and precuneus), were the specific brain regions for identifying CSM. A previous study reported that the overall efficiency and nodal topological properties of the DMN were increased in CSM (8, 48). Our findings suggested that the changes in cognitive networks in CSM patients could become the characteristic features for the diagnosis of CSM. At present, changes in cognitive networks have rarely been investigated in CSM. In the future, studies should investigate in greater detail how CSM and cognitive networks interact.

Our results revealed the GM volume reduction and deformation in the limbic system, including the ACC, MCC, insula, and hippocampus. The ACC and insula are regions associated with chronic pain (50, 51). Cervical spondylosis (CS) patients with neck pain exhibited changes in cortical thickness in the cingulate and insula (34). In our study, the reduced GM volume and structural deformation in the ACC, MCC, and insula indicated that these regions might be involved in pain modulation in CSM patients. Our results were consistent with the modern theories associated with chronic pain structural reorganization (52, 53).

### WM changes in the CSM group correlated with the JOA score

As described above, the alterations of GM structures in CSM in our study indicated extensive damage to the cortices and sub-cortices due to long-term chronic and incomplete SCI. To confirm WM alterations accompanied by GM changes, we further analyzed the volume alterations and deformation degree of WM.

Our results revealed the WM volume reduction and deformation in the ACR, pars genu, and body of the CC. The reduced WM volume in the ACR was positively correlated with neurological dysfunction, as evaluated by the JOA score. Moreover, the WM volume reduction and deformation regions were confirmed to identify CSM in ROI-based MVPA. ACR was associated with the corticospinal tract and the internal capsule (54, 55). The WM volume reduction and deformation of the ACR further confirmed the reorganization of motor function in CSM patients. CC interconnected the bilateral cerebral hemispheres and was key to transmitting cognitive, sensory, and motor information (56). A study has shown that the CC was organized by the anterior callosal fibers that connected frontal regions such as the sensorimotor and PFC (57). In their study, Cunningham et al. found that fractional anisotropy (FA) decreased within the CC in SCI patients and that decreased FA in the CC was a biomarker associated with recovery of upper extremity motor function recovery (55). The WM volume reduction and deformation in ACR and CC in this study suggested that WM integrity was damaged in the frontal cortex and the corticospinal tract in CSM patients.

### Similarities and differences in structural alterations detected by VBM and TBM

Although VBM and TBM were both neuroanatomical algorithms at the voxel level, they focused on diverse aspects of exploring brain structural information. The VBM algorithm is a voxel-based comparison of tissue segmentation maps normalized to the same stereotactic space (14, 58). The TBM algorithm analyzed positional differences between each voxel of the individual brain and the standard template (14, 59). VBM is more sensitive and powerful in uncertain registration (58, 60). TBM reflects subtle local differences in brain structure and is more accurate for uncertainty registration (15, 58, 60–62). The present study found that the combination of VBM and TBM can objectively and completely reflect morphological changes while improving alignment accuracy at the same time.

Moreover, the regions of reduction and deformation were detected in both VBM and TBM, suggesting that these regions were mainly associated with a reduction in GM or WM volume, accompanied by significant local structural deformation. We also observed differences in the changes in various brain structures. The main GM structural changes were detected only by VBM, including the M1, inferior frontal opercularis, lingual gyrus, and MOG. In TBM, some subcortical regions, especially the thalamus, were detected. The differences in these regions may be associated with the discrepancy in the registration accuracy of TBM and VBM, further demonstrating that the sensitivity of TBM and VBM was different in different brain structural alterations. From the biological point of view, the atrophic regions detected by VBM might represent reduced cortical thickness and cerebral surface areas, accompanied by lower local deformation presented by gross volume. TBM detected significant deformation in the subcortical region, possibly related to more subtle anatomical structures. VBM detected larger WM ranges with reduced volume, while TBM detected only one cluster of atrophic WM, entirely included within the VBM-detected areas. This indicated that VBM was more sensitive to detecting structural changes in WM. Bernabéu-Sanz et al. (12) reported the effect of CS in the brain among 27 patients and 24 HCs. CS patients had significant clusters of gray matter loss in the major sensorimotor cortices and the thalamus, especially in the occipital nucleus. The fMRI results showed that the activated motor regions of CS were located on the periphery of the gray matter loss clusters.

### Classification at the whole-brain level

Machine learning techniques sensitively consider the pattern information and have unparalleled power to identify subtle differences in the spatial pattern of functional alterations between diseased and disease-free states (18, 63). Few studies have used machine learning techniques to identify neuroanatomical biomarkers of CSM, and this study was conducted using MVPA. Our results indicated that GM-VBM had better discriminability than other structural measurement methods. A previous multimodal MRI study suggested that DTI was the main measurement to distinguish CSM patients from HCs based on brain structure (12). Our findings complemented the structural recognition ability of machine learning techniques to diagnose CSM. In addition, our MVPA results further indicated that different morphological measurement methods could help discriminate CSM patients from HCs.

Most previous studies used a single structural measurement method to estimate the differences between CSM patients and HCs. Our univariate analyses proved that different measurement methods can provide different information. In our MVPA study, the combination of four structural measurement methods increased the classification accuracy to 81.58%, thus suggesting that the fusion of information from different structural measurement methods can enhance classification performance. The classification accuracy of the fusion was higher than that of the single structural measurement method, indicating that different measurement methods should be considered in the diagnosis of CSM from a neuroimaging diagnostic perspective.

We conducted a diagnostic model to identify characteristic brain impairments in CSM patients based on VBM and TBM with SVM. Our findings showed that the VBM and TBM with SVM performed well in terms of diagnostic performance for identifying characteristic brain impairment in CSM patients compared with XGBoost and LightGBM. It is beneficial for clinicians to identify high-risk groups as early as possible and to formulate intervention strategies in time to improve patient prognosis. The application of machine learning can assist with case triage and diagnoses, enhance image scanning and segmentation, support decision-making, and predict the risk of disease. Future applications may also bring forth inexpensive forms of medical imaging and affordable medical examinations, potentially ending health disparities and creating more accessible services for countries and lower-income populations.

Several limitations should be considered when explaining our findings. First, non-harmonized/different scanners/sequences were used in the retrospective study. While scanner type was regressed as a nuisance variable, GM/WM contrast is inherent to the techniques used, and the robustness of VBM/TBM with respect to MR sequences/scanner differences was not validated experimentally. Second, the behavioral metrics (outside of JOA and motor-related regions in the brain), such as cognition and visual, were not recorded, and the detected morphological changes may be associated with behavioral differences (outside of JOA and motor-related regions in the brain). Third, the degree of cord compression was not measured. Fourth, JOA subscores, including the motor function of the upper/lower extremities, sensory function, and bladder function, were not recorded in the retrospective study, and the image correlation of JOA subscores was not tested. Fifth, both VBM-GM and VBM-WM images from each participant were spatially smoothed using an isotropic Gaussian kernel with a 6-mm FWHM. Previous studies reported kernel sizes of 8 mm and 10 mm (64, 65). Overall, compared with the 6 mm kernel size, no significant differences were found in these sizes. Silver et al. (66) reported that small kernel sizes may be associated with larger (false positive) cluster sizes. Future studies are needed to further validate our results with a prospective design, multiple centers, and a large sample size.

## Conclusion

The present study used univariate and multivariate analyses to delineate widespread characteristic brain structural impairments detected by VBM and TBM in patients with CSM, providing novel brain-based diagnostic biomarkers. The complementary information obtained by VBM and TBM improved the diagnostic accuracy of CSM by examining characteristic structural brain changes. It is possible to develop a neuroanatomic tool for the objective brain-based diagnosis of CSM.

## Data availability statement

The raw data supporting the conclusions of this article will be made available by the authors, without undue reservation.

## Ethics statement

The studies involving humans were approved by Tianjin Medical University General Hospital (Tianjin, China) (No. IRB2023-WZ-065). The studies were conducted in accordance with the local legislation and institutional requirements. The participants provided their written informed consent to participate in this study.

## Author contributions

YW: Conceptualization, Funding acquisition, Investigation, Project administration, Supervision, Writing – original draft, Writing – review & editing. RZ: Conceptualization, Funding acquisition, Investigation, Project administration, Supervision, Writing – original draft, Writing – review & editing. DZ: Data curation, Formal analysis, Methodology, Software, Validation, Writing – review & editing. XF: Data curation, Formal analysis, Methodology, Software, Validation, Writing – review & editing. FS: Data curation, Formal analysis, Methodology, Software, Validation, Writing – review & editing. YC: Data curation, Formal analysis, Methodology, Software, Validation, Writing – review & editing. JM: Data curation, Formal analysis, Methodology, Software, Validation, Writing – review & editing. XG: Data curation, Formal analysis, Methodology, Software, Validation, Writing – review & editing. JZ: Conceptualization, Methodology, Visualization, Writing – review & editing. YX: Conceptualization, Methodology, Visualization, Writing – original draft.
