# Supplementary material for: Voxel- and tensor-based morphometry with machine learning techniques identifying characteristic brain impairment in patients with cervical spondylotic myelopathy
Source: Front Neurol. 2024 Feb 14;15:1267349. doi: 10.3389/fneur.2024.1267349 (PMC10899699; doi:10.3389/fneur.2024.1267349)
Supplement: Supplementary file 1 [file Data_Sheet_1.docx]

**Supplementary Materials**

**Materials and Methods**

**Multivariate Pattern Classification Analysis**

**The MVPA process for GM-VBM, WM-VBM, GM-TBM, and WM-TBM**

The recruited CSM patients and HCs were divided into 57 folds as follows: the 1^st^ patient and the corresponding HC were assigned to the first fold…, by parity of reasoning, the 57^th^ patient and corresponding HC were assigned to the 57^th^ fold. Fifty-six folds were used as the training set, and 1-fold was used as the testing set in each cross-validation. The leave-one-fold-out cross-validation procedure obtained the average classification accuracy of all folds. The interference between the different scanners was removed when the cross-validation procedure was performed. To observe whether scanner differences significantly affected the results, we also executed cross-validation without removing the interference between the different scanners. A feature selection procedure based on weights embedded in the process of cross-validation was implemented as follows: first, all features were acquired by training the classification using the training set; then, the resultant feature weights were sorted from high to low, and the predefined top percentage features with the highest weights were selected; the top features were used to train a new classifier based on the training set, after which the classifier was examined by the testing set; finally, for this cross-validation step, classification accuracy was obtained. Based on the method described above, we obtained classification accuracy from each cross-validation step. The average accuracy was calculated according to all classification accuracies across cross-validation. For selected features, the procedure was repeated in 10% increments from 10% to 100%. Thus, 10 selected feature sets and 10 averaged classification accuracies were obtained.

**The MVPA process for fusion**

The decision values from the classifiers generated by the four measurement methods were obtained from a particular percentage in feature selection (the feature selection for maximum classification accuracy). Then, the four decision values for each test sample generated by the four classifiers were averaged, and the final decision for the given test sample was made based on the sign of the averaged decision values. The final classification accuracy through data fusion was subsequently calculated as the accuracy rate of all test samples.

**Bias-field corrected image CNR values (mean/SD) for CTRL v CSM**

We selected 10 ROIs in three different structures of central nervous system based on the literature [1]. For the WM, the ROIs were placed in the left and right centrum semiovale, in the genu and splenium of corpus callosum. For the GM, the ROIs were placed in the left and right frontal and occipital cortex. For cerebrospinal fluid (CSF), the ROIs were positioned in left and right anterior horn of the lateral ventricles. Mean and standard deviation of the signal intensity were calculated in each ROI. For the left and the right ROIs placed in the same structures (e.g. left and right frontal cortex), the mean value of the ROIs was calculated to represent the structure (e.g. frontal cortex) as the mean value is the better estimation to represent the differences between the two hemispheres. Finally, the CNR was calculated. CNR were only assessed for different type of structures. Manually outline the above ROIs. The diameter of ROI was 4mm. CNR was assessed in the CSM and HCs by using the following formula:

CNR=∣I_A_-I_B_∣/σ

where I_A_ and I_B_ are the mean signal intensity in two different structures (structures in WM and GM, WM and CSF, GM and CSF)

σ is the standard deviation of all 10 ROIs.

| CNR | 3D MPRAGE | 3D BRAVO |
| --- | --- | --- |
| CSM |  |  |
| WM-GM | 20.36±3.77 | 10.82±5.16 |
| GM-CSF | 18.25±4.00 | 25.81±6.71 |
| WM-CSF | 38.62±3.42 | 47.07±5.55 |
| HCs |  |  |
| WM-GM | 12.96±7.78 | 19.54±6.60 |
| GM-CSF | 29.45±4.03 | 35.38±4.35 |
| WM-CSF | 42.41±8.48 | 58.91±10.21 |

**Bias-field corrected image SNR values (mean/SD) for GE v Siemens**

| Sequence | SNR |
| --- | --- |
| 3D MPRAGE | 81.34±15.64 |
| 3D BRAVO | 198.55±37.65 |

[1] Di Giuliano F, Minosse S, Picchi E, Ferrazzoli V, Da Ros V, Muto M, Pistolese CA, Garaci F, Floris R. Qualitative and quantitative analysis of 3D T1 Silent imaging. Radiol Med. 2021 Sep;126(9):1207-1215. doi: 10.1007/s11547-021-01380-6.

**Table S1** Demographic and clinical characteristics of participants

| Characteristics | CSM patients (n=57) | HCs (n=57) | *P*-value |
| --- | --- | --- | --- |
| Age, years, Mean ± SD | 52.7±12.4 | 50.9±13.6 | 0.315 |
| Sex (male/female)  Education level, years, Mean ± SD | 34/23  10.8±2.8 | 31/26  11.6±3.2 | 0.705  0.146 |
| Preoperative JOA score, Mean ± SD | 11.6±1.7 | - | - |

CSM, cervical spondylotic myelopathy; HCs, healthy controls; Mean ± SD, Mean ± standard deviation; JOA, Japanese Orthopedic Association.

*P*-values of age and education level were respectively calculated with the two-sample t-test and sex was calculated with χ2 tests.

**Table S2** GM volume reductions detected by VBM in CSM patients compared with HCs

| **Cluster No.** | **Brain region** | **Peak MNI coordinates** | ***t* value** | **Cluster size (voxels)** |
| --- | --- | --- | --- | --- |
|  |  | **x y z** |  |  |
| 1 | middle temporal gyrus, fusiform gyrus, L insula, cerebellum, vermis, L postcentral gyrus, superior temporal gyrus, temporal pole, inferior parietal lobule, hippocampus, L Rolandic operculum, L angular gyrus, lingual gyrus, L putamen, L middle occipital gyrus, L inferior orbitofrontal cortex, L inferior frontal gyrus pars opercularis, L precentral gyrus | -69 -45 4.5 | -5.78 | 33711 |
| 2 | R postcentral gyrus, R precentral gyrus, R supramarginal gyrus | 42 -19.5 64.5 | -4.83 | 2737 |
| 3 | middle cingulum, L anterior cingulum | -3 9 39 | -4.50 | 3095 |
| 4 | R middle frontal gyrus, R inferior frontal gyrus pars triangularis, R premotor cortex, R inferior frontal gyrus pars opercularis | 36 9 54 | -4.38 | 1997 |
| 5 | inferior orbitofrontal cortex, R insula, medial orbital gyrus, R Rolandic operculum, middle orbitofrontal cortex, superior orbitofrontal cortex, L middle frontal gyrus, R gyrus rectus, medial superior frontal gyrus, inferior frontal gyrus pars triangularis, L anterior cingulum, R caudate, R amygdala, R putamen, R superior temporal gyrus, R parietal operculum | 40.5 40.5 -10.5 | -5.85 | 21653 |

VBM, voxel-based morphometry; CSM, cervical spondylotic myelopathy; HCs, healthy controls; MNI, Montreal Neurological Institute; L, left; R, right.

The threshold for statistical significance was set at FDR-corrected *P* < 0.05. AAL atlas was used to define the brain regions. Age, sex, scanner type, and total intracranial volume (TIV) were regressed out as covariates.

**Table S3** WM volume reductions detected by VBM in CSM patients compared with HCs

| **Cluster No.** | **Brain region** | **Peak MNI coordinates** | ***t* value** | **Cluster size (voxels)** |
| --- | --- | --- | --- | --- |
|  |  | **x y z** |  |  |
| 1 | R anterior corona radiata, corpus callosum | 28.5 34.5 -4.5 | -4.46 | 1110 |
| 2 | L anterior corona radiata | -16.5 25.5 13.5 | -4.57 | 739 |

VBM, voxel-based morphometry; CSM, cervical spondylotic myelopathy; HCs, healthy controls; MNI, Montreal Neurological Institute; L, left; R, right.

The threshold for statistical significance was set at FDR-corrected *P* < 0.05. AAL atlas was used to define the brain regions. Age, sex, scanner type, and total intracranial volume (TIV) were regressed out as covariates.

**Table S4** GM deformation detected by TBM in CSM patients compared to HCs

| **Cluster No.** | **Brain region** | **Peak MNI coordinates** | ***t* value** | **Cluster size (voxels)** |
| --- | --- | --- | --- | --- |
|  |  | **x y z** |  |  |
| 1 | middle temporal gyrus, fusiform gyrus, vermis, cerebellum, putamen, hippocampus, thalamus, R superior temporal gyrus, amygdala, R para-hippocampal gyrus, L pallidum | 0 -64.5 -22.5 | -4.80 | 18610 |
| 2 | R inferior orbitofrontal cortex, superior orbitofrontal cortex, R middle orbitofrontal cortex, medial orbital gyrus, R putamen, gyrus rectus, middle frontal gyrus, L anterior cingulum, R insula, medial superior frontal gyrus, middle cingulum, R inferior frontal gyrus pars triangularis, R caudate, R Rolandic operculum, R amygdala | 19.5 36 -18 | -5.37 | 15367 |
| 3 | L superior frontal gyrus, L middle frontal gyrus, L inferior frontal gyrus pars triangular | -13.5 52.5 28.5 | -4.15 | 1395 |
| 4 | L middle temporal gyrus, L inferior parietal lobule, L inferior temporal gyrus, L postcentral gyrus | -57 -64.5 0 | -5.13 | 2539 |

TBM, tensor-based morphometry; CSM, cervical spondylotic myelopathy; HCs, healthy controls; MNI, Montreal Neurological Institute; L, left; R, right.

The threshold for statistical significance was set at FDR-corrected *P* < 0.05. AAL atlas was used to define the brain regions. Age, sex, and scanner type were regressed out as covariates.

**Table S5** WM deformation detected by TBM in CSM patients compared to HCs

| **Cluster No.** | **Brain region** | **Peak MNI coordinates** | ***t* value** | **Cluster size (voxels)** |
| --- | --- | --- | --- | --- |
|  |  | **x y z** |  |  |
| 1 | R anterior corona radiata | 28.5 31.5 -1.5 | -4.80 | 763 |

TBM, tensor-based morphometry; CSM, cervical spondylotic myelopathy; HCs, healthy controls; MNI, Montreal Neurological Institute.

The threshold for statistical significance was set at FDR-corrected *P* < 0.05. AAL atlas was used to define the brain regions. Age, sex, and scanner type were regressed out as covariates.

**Table S6** Significant regions at the ROI-based MVPA

| **Overlapping between VBM and TBM** | **Nonoverlapping in VBM** | **Nonoverlapping in TBM** |
| --- | --- | --- |
| premotor area (BA6) | PFC (lateral area 10) | PFC (area 13, dorsal area 9/46, opercular and ventral area 44, orbital area 12/47, medial area 14) |
| somatosensory cortex (BA1, 2, 3) | cingulate cortex (ACC (rostroventral and caudodorsal area 24, subgenual area 32)) | insula (dorsal agranular and insula granular, ventral dysgranular and granular insula) |
| M1 (BA4) | STG (TE1.0 and TE1.2) | MTG (dorsolateral area 37) |
| PFC (area 46) | IPL (A40rv, rostroventral area 40 (PFop)) | ITG (caudolateral of area 20) |
| insula (ventral agranular insula) | insula (dorsal dysgranular insula) | superior parietal lobule (lateral area 5) |
| precuneus (area 31) | occipital cortex (occipital pole, IOG, medioventral occipital cortex) | IPL (caudal and rostroventral area 39) |
| hippocampus (rostral hippocampus) | BG (nucleus accumbens) | precuneus (dorsomedial parietoccipital sulcus) |
| BG (medial amygdala, ventral caudate) | thalamus (lateral prefrontal thalamus) | MOG |
| thalamus (medial prefrontal thalamus, sensory thalamus) | cerebellum (I-IV, VIIIb, IX) | BG (globus pallidus, dorsolateral putamen, dorsal caudate) |
| cerebellum (Crura II, V) |  | thalamus (lateral prefrontal thalamus, premotor thalamus) |
|  |  | cerebellum (Vermis VI) |
| right ACR | left ACR and bilateral genu of CC and right body of CC |  |

ROI: regions of interest; MVPA, multivariate pattern analysis; VBM, voxel-based morphometry; TBM, tensor-based morphometry; M1, primary motor cortex; BG, basal ganglia; PFC, prefrontal cortex; ACC, anterior cingulate cortex; STG, superior temporal gyrus; IPL, inferior parietal lobule; IOG, inferior occipital gyrus; MTG, middle temporal gyrus; ITG, inferior temporal gyrus; MOG, middle occipital gyrus; ACR, anterior corona radiata; CC, corpus callosum.

Statistical significance was thresholded at FDR-corrected *P* < 0.05.

**Fig S1**


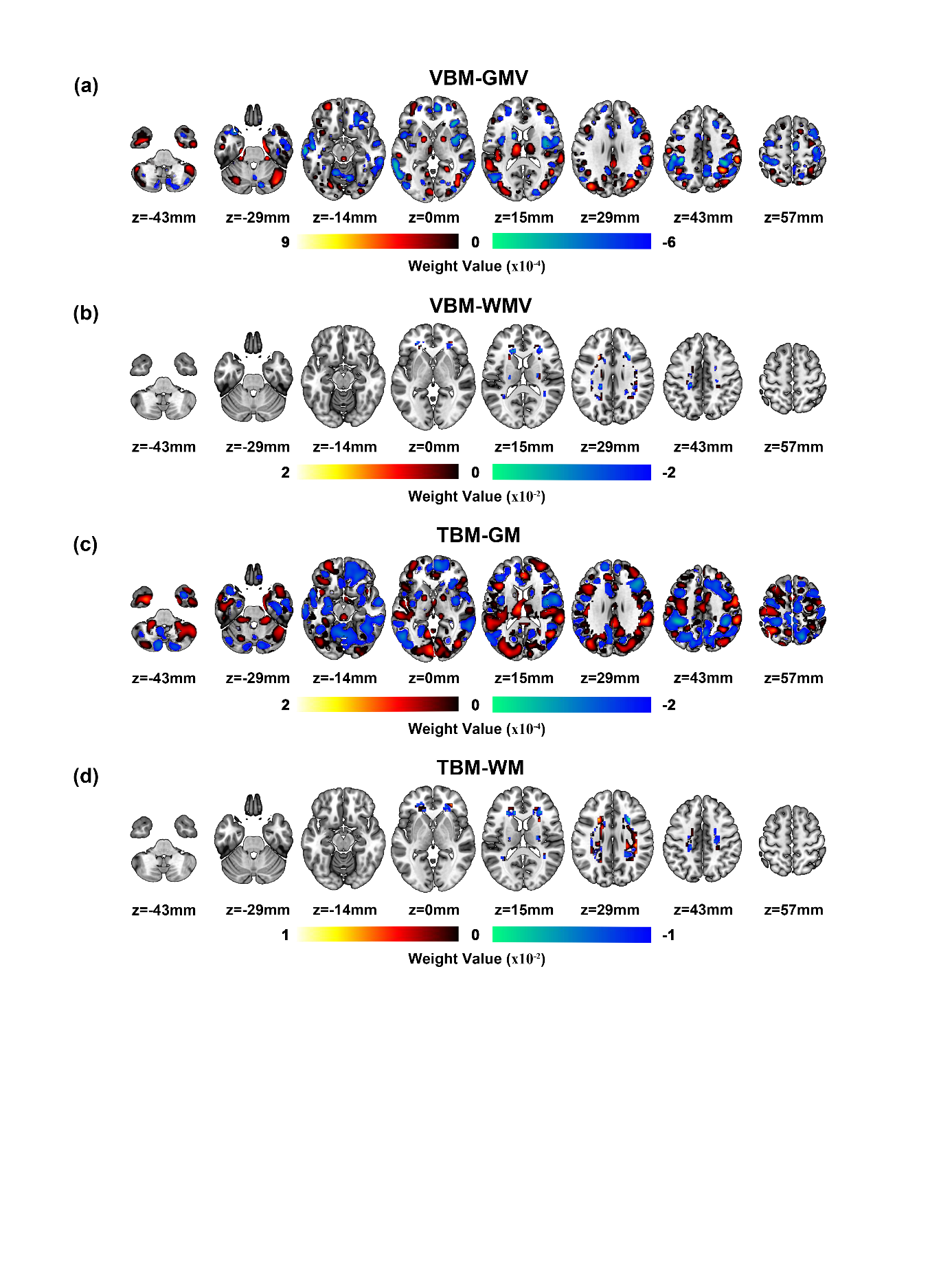


Fig S1: The weight maps of the highest classification accuracy of features from each imaging measure that contributed to the fusion. (a) GM-VBM; (b) WM-VBM; (c) GM-TBM; (d) WM-TBM. The scanner type was regressed as a covariate.

**Fig S2**


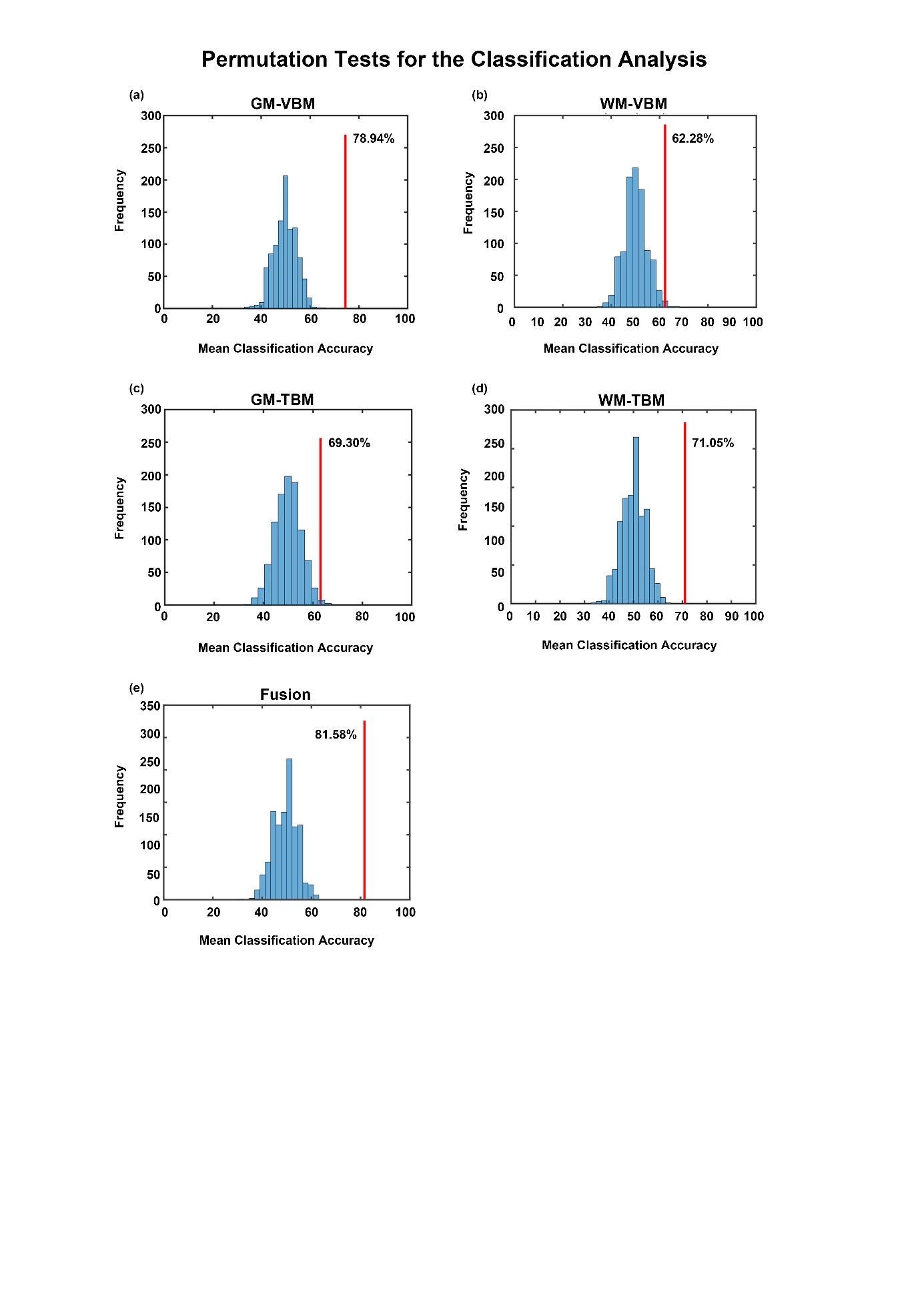


Fig S2: Classification accuracy in the discrimination of CSM patients from HCs, along with the corresponding null distributions. (a-d) GM-VBM, WM-VBM, GM-TBM, WM-TBM measurements; (e) Fusion measurements. Classification accuracy levels are presented as red vertical lines, and null distributions (based on 1000 permutations) are illustrated by bell-shaped distributions centered around a chance accuracy level of 50%. The *P*-values were calculated as the number of generated permutations that exceeded or equaled the actual classification accuracy divided by the total number of permutations. All accuracies were statistically significant (*P* <0.005). The scanner type was regressed as a covariate.

**Fig S3**


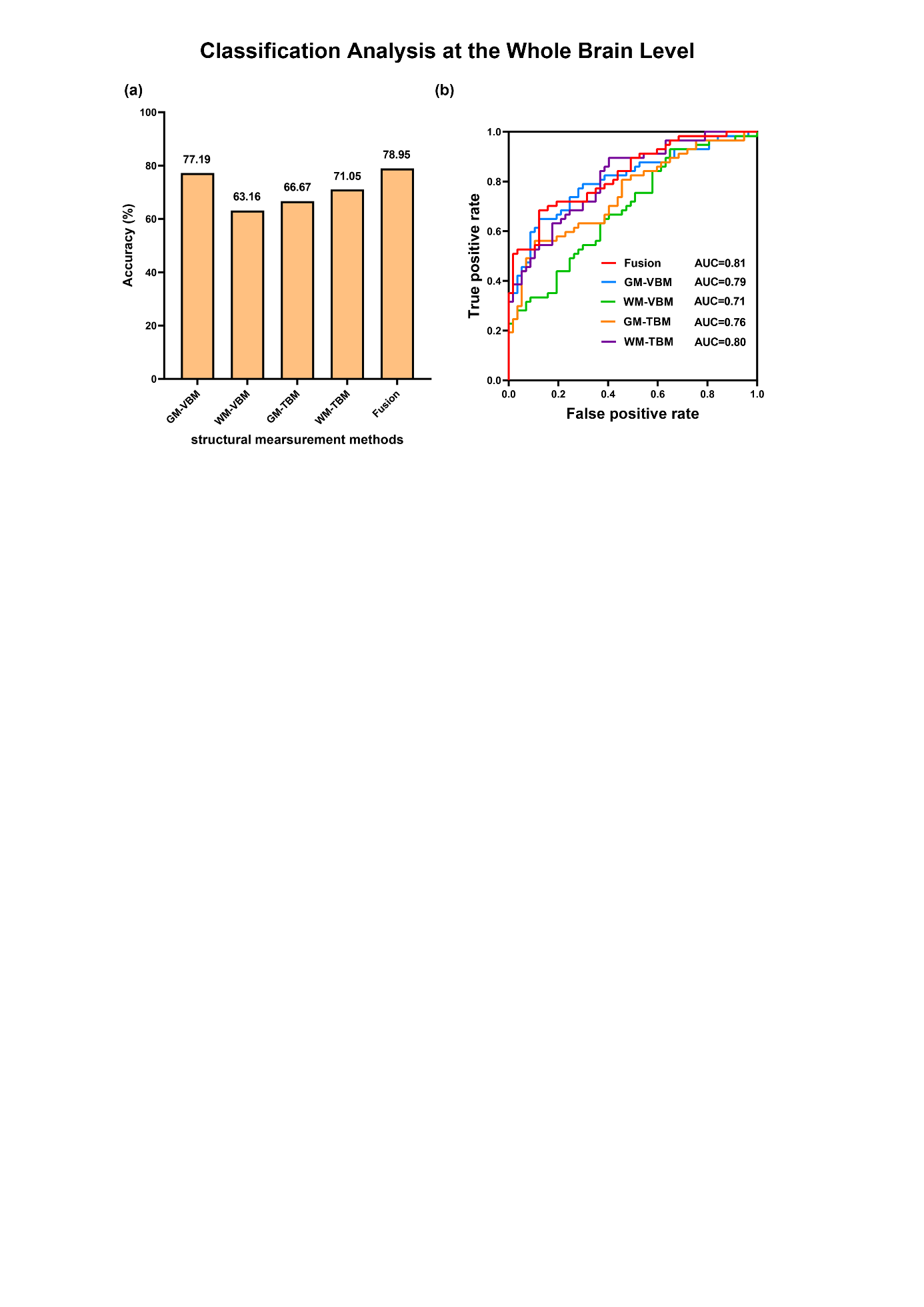


Fig S3: Classification analysis at the whole-brain level based on GM-VBM, WM-VBM, GM-TBM, WM-TBM, and fusion. (a) Classification accuracy for each structural measurement method and the combined measurement method (fusion). (b) Receiver operating characteristic (ROC) curves and corresponding AUCs for each structural measurement method and the combined measurement method (fusion). The scanner type was not regressed as a covariate.

**Fig S4**


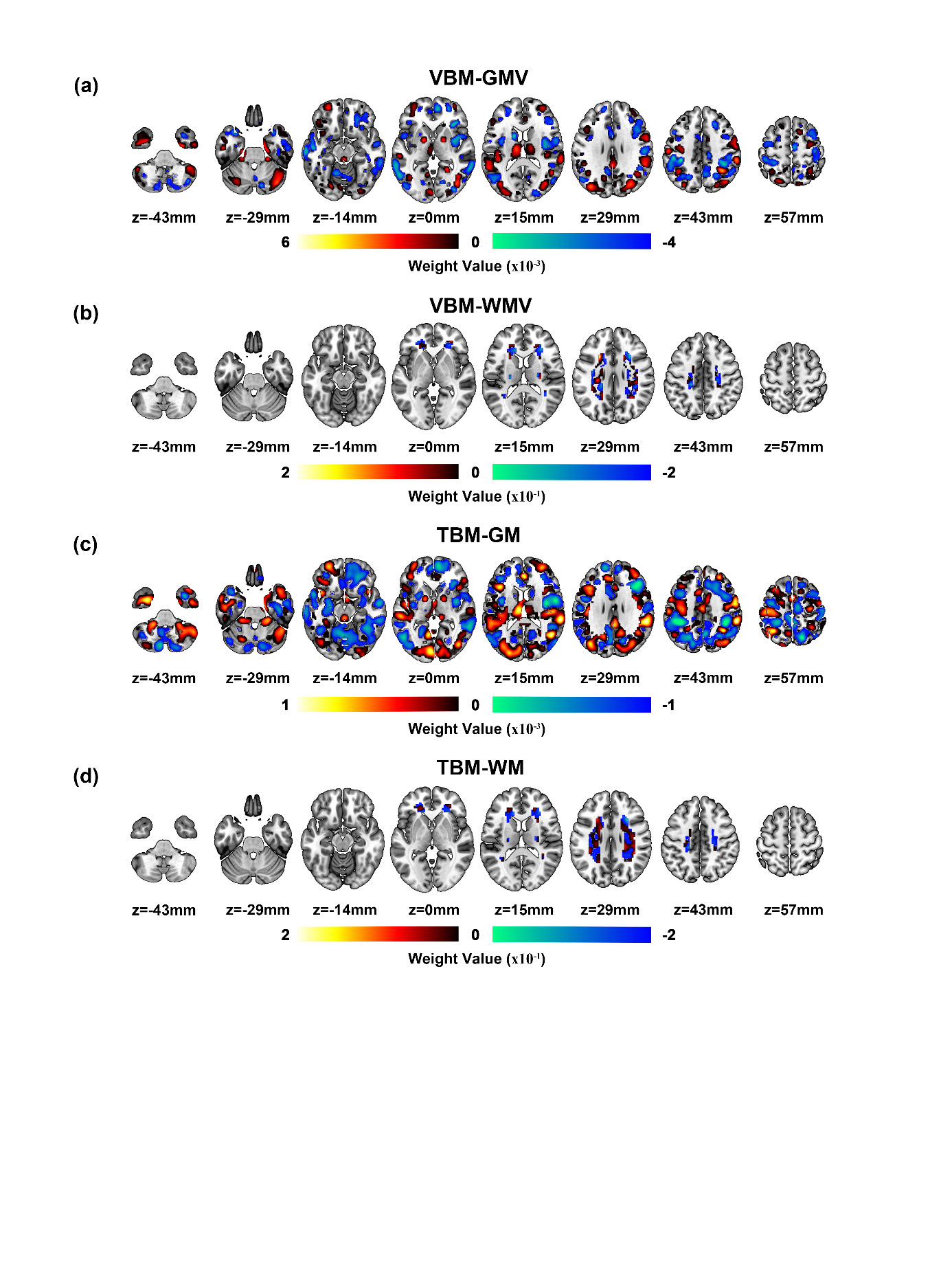


Fig S4: The weight maps of the highest classification accuracy of features from each imaging measure that contributed to the fusion. (a) GM-VBM; (b) WM-VBM; (c) GM-TBM; (d) WM-TBM. The scanner type was not regressed as a covariate.

**Fig S5**


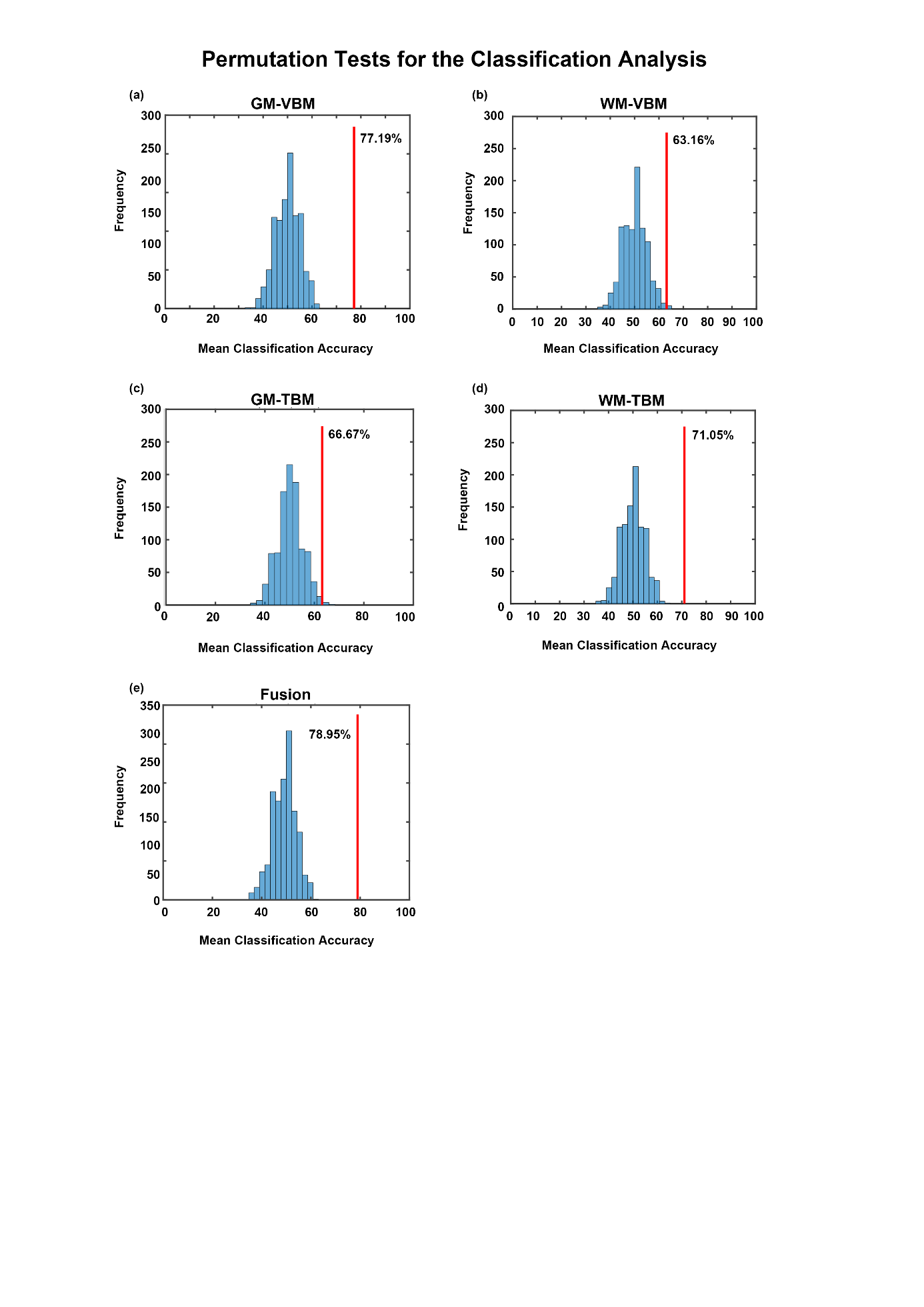


Fig S5: Classification accuracy in the discrimination of CSM patients from HCs, along with the corresponding null distributions. (a-d) GM-VBM, WM-VBM, GM-TBM, and WM-TBM measurement; (e) fusion measurement. Classification accuracy levels are presented as red vertical lines, and null distributions (based on 1000 permutations) are illustrated by bell-shaped distributions centered around a chance accuracy level of 50%. The *P*-values were calculated as the number of generated permutations that exceeded or equaled the actual classification accuracy divided by the total number of permutations. All accuracies were statistically significant (*P* <0.005). The scanner type was not regressed as a covariate.
